# Chemoradiotherapy is an alternative choice for patients with primary mediastinal seminoma

**DOI:** 10.1186/s13014-022-02013-6

**Published:** 2022-03-26

**Authors:** Yirui Zhai, Bo Chen, Xiaoli Feng, Kan Liu, Shulian Wang, Zhouguang Hui, Qinfu Feng, Junling Li, Zefen Xiao, Jima Lv, Yushun Gao, Yueping Liu, Hui Fang, Jianyang Wang, Lei Deng, Wenyang Liu, Wenqing Wang, Zongmei Zhou, Ye-Xiong Li

**Affiliations:** 1 Department of Radiation Oncology, National Cancer Center/National Clinical Research Center for Cancer/Cancer Hospital, Chinese Academy of Medical Sciences and Peking Union Medical College, Beijing, China; 2 Department of Pathology, National Cancer Center/National Clinical Research Center for Cancer/Cancer Hospital, Chinese Academy of Medical Sciences and Peking Union Medical College, Beijing, China; 3 Department of Radiology, National Cancer Center/National Clinical Research Center for Cancer/Cancer Hospital, Chinese Academy of Medical Sciences and Peking Union Medical College, Beijing, China; 4 Department of VIP Medical Service, National Cancer Center/National Clinical Research Center for Cancer/Cancer Hospital, Chinese Academy of Medical Sciences and Peking Union Medical College, Beijing, China; 5 Department of Medical Oncology, National Cancer Center/National Clinical Research Center for Cancer/Cancer Hospital, Chinese Academy of Medical Sciences and Peking Union Medical College, Beijing, China; 6 Department of Thoracic Surgery, National Cancer Center/National Clinical Research Center for Cancer/Cancer Hospital, Chinese Academy of Medical Sciences and Peking Union Medical College, Beijing, China

**Keywords:** Chemotherapy, Germ cell carcinoma, Mediastinal seminoma, Radiotherapy, Surgery

## Abstract

**Background:**

The low incidence of primary mediastinal seminomas has precluded the development of clinical trials on mediastinal seminomas. We investigated the clinicopathologic characteristics, prognosis of patients with primary mediastinal seminomas as well as the efficiency of nonsurgical treatments compared with treatments containing surgery.

**Methods:**

We retrospectively collected data on the clinicopathologic characteristics, treatments, toxicities, and survival of 27 patients from a single center between 2000 and 2018. Patients were divided into two groups according to whether they received operation. Survivals were assessed using the Kaplan–Meier method. Univariate analysis was performed using the log-rank test.

**Results:**

The median age was 28 (13–63) years. The most common symptoms were chest pain (29.6%), cough (25.9%), and dyspnea (22.2%). There were 13 and 14 patients in surgery and non-surgery group. Patients in the non-surgical group were more likely to be with poor performance scores (100% vs. 76.9%) and disease invaded to adjacent structures (100% vs. 76.9%) especially great vessels (100% vs. 46.2%).The median follow-up period was 32.23 (2.7–198.2) months. There was no significant difference of overall survival (5-year 100% vs. 100%), cancer-specific survival (5-year 100% vs. 100%), local regional survival (5-year 91.7% vs. 90.0%, *p* = 0.948), distant metastasis survival (5-year 90.9% vs. 100.0%, *p* = 0.340) and progression-free survival (82.5% vs. 90.0%, *p* = 0.245) between patients with and without surgery.

**Conclusions:**

Primary mediastinal seminoma was with favorable prognosis, even though frequently invasion into adjacent structures brings difficulties to surgery administration. Chemoradiotherapy is an alternative treatment with both efficacy and safety.

## Introduction

Primary mediastinal germ-cell neoplasms are rare neoplasms. Mediastinal seminoma accounts for approximately 10–16% of mediastinal germ-cell neoplasms and 0.5–5% of all mediastinal tumors [1, 2]. This low incidence has precluded the development of randomized clinical trials on mediastinal seminoma, and present knowledge is based on case reports and very small studies, mostly with sample sizes of 1–16 patients. Furthermore, previous studies included patients with other germ-cell subtypes despite the many distinctive features of seminomas and non-seminomas [3–5], and therefore it is difficult to draw definite conclusions from those studies.

Although complete resection has been considered and delivered as a predominant treatment, the absence of symptoms at very beginning leads to disease diagnosis at a more advanced stage and increased the difficulties of resection. For these patients, non-surgical treatments including radiation and chemotherapy are recommended alternatively. However, the efficacy of these therapeutic methods is unclear.

Thus, in this study, we investigated the clinicopathologic characteristics, prognosis of patients with primary mediastinal seminomas as well as the efficiency of nonsurgical treatments compared with treatments containing surgery.

## Methods

### Patient selection

This retrospective study was approved by the institutional ethics committee of our institution. Informed written consent including the therapeutic regimens and possible data collections for the future academic analysis was obtained from patients before the treatment. We examined patients with primary mediastinal seminoma with the complete medical reports treated at the National Cancer Center, Beijing, China, between January 2000 and December 2018.

### Clinicopathologic variables

Patients who fulfilled the enrolment criteria were classified into surgical group and non-surgical group according to the primary treatment they received. Data regarding patient demographics, symptoms, tumor size, history of smoking and alcohol use, invasion status, treatment protocols, and survival for each group and the whole population were collected. For all cases, physical examination and chest and abdominal computed tomography (CT) were performed before treatment. Ultrasound of the testicles was also performed in all male patients to rule out gonadal involvement.

The sporadic incidence of primary mediastinal seminomas has also contributed to the preclusion of development of a staging system. To describe the extent of invasion and explore the prognosis of patients with primary mediastinal seminoma, we adopted the Masaoka staging system, which is widely used for another mediastinal tumor, that is, thymic neoplasms [6]. To characterize the invasive sites of the mediastinal seminoma, we reviewed the primary CT scans and described the invasive regions according to the International Association for the Study of Lung Cancer (IASLC) mediastinal lymph node system [7].

### Outcomes and statistical analyses

Tumor response was initially assessed by a senior radiologist and a radiation oncologist and then confirmed by certain investigators for 1 month after treatment, according to the Response Evaluation Criteria in Solid Tumors (RECIST), version 1.1. Treatment toxicities were graded according to the National Cancer Institute Common Terminology Criteria for Adverse Events version 4.0. Overall survival (OS) was defined as the time from diagnosis to death, and progression-free survival (PFS) was defined as the time from diagnosis to disease progression or death. Local–regional relapse-free survival (LRFS) was defined as the time from diagnosis to local–regional recurrence, whereas distant metastasis-free survival (DMFS) was defined as the time from diagnosis to any new distant metastasis. Cancer-specific survival (CSS) was defined as the time from diagnosis to cancer-induced death. Survival curves were plotted using the Kaplan–Meier method.

The characteristics of patients in two groups were compared using chisquare test. Univariate analysis was performed using the log-rank test and included surgery and the following variables: Eastern Cooperative Oncology Group performance status score (ECOG PS), sex, age, Masaoka stage, histology, great vessel (aorta, pulmonary artery, pulmonary vein, or brachiocephalic vein) invasion, R0 resection, radiotherapy, and chemotherapy; *p* < 0.05 was considered statistically significant. All statistical analyses were conducted using SPSS 23.0 software (IBM Corp., Armonk, NY).

## Results

### Number of patients

We identified 30 patients with a pathologic diagnosis of mediastinal seminoma in the database. However, one patient was excluded because of incomplete data and two were excluded for having mixed germ-cell neoplasms. Thus, 27 patients were finally included. For these 27 patients, 13 patients and 14 patients enrolled surgical group and nonsurgical group.

### Clinical characteristics

The median age of the whole group was 28 (13–63) years. The median maximum primary tumor diameter was 9.9 (3.3–15) cm. The most common symptom was chest pain. Station 3A was the most common site of invasion. Adjacent tissue invasion was also very common. Most patients were diagnosed with Masaoka stage III–IV disease. Patients in the non-surgical group were more likely to be with poor performance scores and disease invaded to adjacent structures especially great vessels. Details of patient characteristics as well as the comparison of the two groups were listed in Table 1.

**Table 1:** Patients’ characteristics

Characteristic	All N (%)	NS N (%)	S N (%)	*p*	Characteristic	All N (%)	NS N (%)	S N (%)	*p*
Sex				0.290	Primary tumor invasive site				
Male	26 (96.3)	14 (100)	12 (92.3)		1R	5 (18.5)	4 (28.6)	1 (7.7)	0.163
Female	1 (3.7)	0 (0)	1 (7.7)		1L	5 (18.5)	4 (28.6)	1 (7.7)	0.163
Age, years				0.918	2R	11 (40.7)	9 (64.3)	2 (15.4)	0.010
≤ 18	7 (25.9)	3 (21.4)	3 (23.1)		2L	11 (40.7)	6 (42.9)	5 (38.5)	0.816
> 18	20 (74.1)	11 (78.6)	10 (76.9)		3A	27 (100.0)	14 (100)	13 (100)	1.000
First symptom				0.200	3P	6 (22.2)	5 (35.7)	1 (7.7)	0.080
Dyspnea	6 (22.2)	2 (14.3)	4 (30.8)		4R	11 (40.7)	9 (64.3)	2 (15.4)	0.010
Chest pain	8 (29.6)	5 (35.7)	3 (23.1)		4L	11 (40.7)	6 (42.9)	5 (38.5)	0.816
Cough	7 (25.9)	4 (28.6)	3 (23.1)		5	14 (51.9)	8 (57.1)	6 (46.2)	0.568
Vomit	1 (3.7)	1 (7.1)	0 (0)		6	19 (70.4)	11 (78.6)	8 (61.5)	0.333
Facial edema	2 (7.4)	2 (14.3)	0 (0)		7	4 (14.8)	3 (21.4)	1 (7.7)	0.315
Symptomless	3 (11.1)	0	3 (23.1)		8	0 (0.0)	0 (0.0)	0 (0.0)	1.000
SVCS				0.148	9R	1 (3.7)	1 (7.1)	0 (0.0)	0.326
Yes	10 (37.0)	7 (50.0)	3 (23.1)		9L	2 (7.4)	0 (0.0)	2 (15.4)	0.127
No	17 (63.0)	7 (50.0)	10 (76.9)		10R	1 (3.7)	1 (7.1)	0 (0.0)	0.326
ECOG PS score				0.056	10L	4 (14.8)	1 (7.1)	3 (23.1)	0.244
0	3 (11.1)	0 (0)	3 (23.1)		PDL	2 (7.4)	2 (14.3)	0 (0.0)	0.157
1	24 (88.9)	14 (100)	10 (76.9)		Invaded adjacent tissue				
Alcohol use				0.557	Aorta	15 (55.6)	11 (78.6)	4 (30.8)	0.013
Yes	1 (3.7)	0 (0)	1 (7.7)		PA	8 (29.6)	4 (28.6)	4 (30.8)	0.901
No	26 (96.3)	14 (100)	12 (92.3)		SVC	15 (55.6)	12 (85.7)	3 (23.1)	0.001
Smoking				0.557	PV	3 (11.1)	1 (7.1)	2 (15.4)	0.496
Yes	5 (19.5)	2 (14.3)	3 (23.1)		BV	5 (18.5)	2 (14.3)	3 (23.1)	0.557
No	22 (81.5)	12 (85.7)	10 (76.9)		AV	1 (3.7)	1 (7.1)	0 (0.0)	0.326
Maximum diameter, cm				0.228	CA	2 (7.4)	1 (7.1)	1 (7.7)	0.957
≤ 5	2 (7.4)	0 (0)	2 (15.4)		CV	1 (3.7)	0 (0.0)	1 (7.7)	0.290
5.1–10	14 (51.9)	7 (50.0)	7 (53.8)		Lung	12 (44.4)	6 (42.9)	6 (46.2)	0.863
> 10	11 (40.7)	7 (50.0)	4 (30.8)		Pericardium	13 (48.1)	7 (50.0)	6 (46.2)	0.842
Adjacent tissue invasion				0.056	Heart	2 (7.4)	1 (7.1)	1 (7.7)	0.957
Yes	24 (88.9)	14 (100)	10 (76.9)		Bronchus	1 (3.7)	0 (0.0)	1 (7.7)	0.290
No	3 (11.1)	0 (0)	3 (23.1)		Trachea	1 (3.7)	0 (0.0)	1 (7.7)	0.290
Great vessel invasion				0.001	Sternum	2 (7.4)	2 (14.3)	0 (0.0)	0.157
Yes	20 (74.1)	14 (100)	6 (46.2)		Masaoka stage				0.028
No	7 (25.9)	0 (0)	7 (53.8)		I	2 (7.4)	0 (0.0)	2 (15.4)	
Lymph node metastasis				0.745	II	1 (3.7)	0 (0.0)	1 (7.7)	
Yes	7 (25.9)	4 (28.6)	3 (23.1)		IIIa	4 (14.8)	0 (0.0)	4 (30.8)	
No	20 (74.1)	10 (71.4)	10 (76.9)		IIIb	13 (48.1)	10 (71.4)	3 (23.1)	
Distant metastasis				0.957	IVa	0 (0.0)	0 (0.0)	0 (0.0)	
Yes	2 (7.4)	1 (7.1)	1 (7.7)		IVb	7 (25.9)	4 (28.6)	3 (23.1)	
No	25 (92.6)	13 (92.9)	12 (92.3)						

*AV* azygos vein, *BV* brachiocephalic vein, *CA* carotid artery, *CV* carotid vein, *ECOG PS* Eastern Cooperative Oncology Group performance status, *PA* pulmonary artery, *PDL* pericardial diaphragmatic lymph node, *PV* pulmonary vein, *SVC* superior vena cava, *SVCS* superior vena cava syndrome

### Laboratory and immunohistochemical characteristics

Human chorionic gonadotropin (hCG) and lactate dehydrogenase (LDH) levels were assessed in all patients and 16 and 11 patients had the data before treatment and the respective observed values were 21.48 (0.2–900.0) IU/mL and 226 (0.68–1029) ng/mL. Among them, hCG and LDH levels increased in 14 and 5 patients, respectively. After treatment, hCG and LDH levels were reassessed in 22 and 16 patients, respectively. All results were normal, with corresponding median values of < 0.1 IU/mL and 168 (122–240) ng/mL.

Immunohistochemical characteristics evaluated according to different HE expressions to achieve diagnostic accuracy (Table 2) revealed high positivity rates for PLAP, OCT3/4, and SALL4.

**Table 2:** Immunohistochemical results

Antibody	No. of patients		Antibody	No. of patients	
	Positive	Negative		Positive	Negative
AE1/AE3	6	10	CK8/18	0	2
AFP	0	8	D2-40	1	1
CD3	0	8	EMA	0	2
CD5	1	3	HMB45	0	3
CD20	0	7	hCG	0	6
CD99	0	1	LCA	0	15
CD30	0	12	Melan-pan	0	2
CD117	14	1	NSE	0	2
CD163	1	0	OCT3/4	5	0
CEA	1	2	P63	0	1
CgA	0	2	PAX5	0	2
ChA	0	2	PLAP	18	0
CK5	0	1	SALL4	6	0
CK7	1	1	Syn	0	4
CK18	2	0	TdT	0	6
CK19	2	3	TTF-1	0	3
CK34βE12	0	1	Vimentin	1	4
CK5/6	0	2			

### Treatment details

Table 3 lists treatment details. Surgery, radiation, and chemotherapy were administered in 13, 16, and 25 patients, respectively. R0 resection were performed in 9 patients. Eleven patients received neoadjuvant or adjuvant chemotherapy and/or chemotherapy. For three-dimensional conformal radiotherapy or more advanced techniques, the following target volume delineation principles were adhered to. The gross tumor volume (GTV) included the primary tumor and was determined by thoracic CT. The clinical target volume (CTV) included the GTV plus a 5-mm margin and regions of invasion. The planning target volume was created by adding an additional 5-mm margin to the CTV. The median radiation dose is 42.3 Gy in the whole group. For patients in non-surgical group, the median radiation dose is 45 Gy (ranged 30–56 Gy). Among the 25 patients who received chemotherapy, 22 received bleomycin, etoposide, and cisplatin (BEP).

**Table 3:** Treatment details

	N	%		N	%
Treatment			Radiation dose (gray)	42.3 (25.2–56.0)	
CHT	2	7.4	Radiation technique		
CRT	12	44.4	2D	4	14.8
CSR	1	3.7	3DCRT	3	11.1
S	2	7.4	IMRT	7	25.9
SCHT	7	25.9	VMAT	2	7.4
SCRT	3	11.1	CHT regimen		
Resection			BEP	22	81.5
R0	9	33.3	EP	1	3.7
R2	4	14.8	CEP	1	3.7
No	14	51.9	PEP	1	3.7
RT			CHT cycle		
Yes	16	59.3	2	2	7.4
No	11	40.7	3	2	7.4
CHT			4	13	48.1
Yes	25	92.6	6	8	29.6
No	2	7.4			

*2D* two-dimensional conformal technique, *3DCRT* three-dimensional conformal radiotherapy, *CHT* chemotherapy, *CRT* chemoradiotherapy, *CSR* chemotherapy, surgery, and radiotherapy, *IMRT* intensity-modulated radiotherapy, *RT* radiotherapy, *S* surgery, *SCHT* surgery plus chemotherapy, *SCRT* surgery plus chemoradiotherapy, *VMAT* volumetric modulated arc therapy

### Toxicities

Grade 3 toxicities were observed in 4 patients (14.8%) (Table 4). Three of them were in non-surgical group and one of them was in surgical group. Meanwhile, grade 3 hematological and nonhematological toxicities were observed in 3 patients (11.1%) and 2 patients (7.4%), respectively (including one patient with both hematological and nonhematological toxicities).

**Table 4:** Toxicities

	Grade 1	Grade 2	Grade 3	Total
RP	5 (18.5)	0	0	5 (18.5)
Esophagitis	1 (3.7)	0 (0.0)	0 (0.0)	1 (3.7)
Dermatitis	11 (40.7)	0 (0.0)	0 (0.0)	11 (40.7)
Vomit	3 (11.1)	5 (18.5)	2 (7.4)	10 (37.0)
Hair loss	6 (22.2)	16 (59.3)	0 (0.0)	22 (81.5)
Leucopenia	10 (37.0)	7 (25.9)	2 (7.4)	19 (71.4)
Neutropenia	8 (29.6)	7 (25.9)	3 (11.1)	18 (66.7)
Anemia	0 (0.0)	1 (3.7)	0 (0.0)	1 (3.7)
Thrombopenia	1 (3.7)	0 (0.0)	0 (0.0)	1 (3.7)

*RP* radiation-induced pneumonitis

### Tumor response

Among the 25 patients who received chemotherapy, 19 achieved a partial response (PR), 1 had stable disease, and 5 had no application since chemotherapy was used as adjuvant therapy. Of the 16 patients who received radiotherapy, 2 and 14 patients achieved complete response (CR) and PR, respectively. After all treatments, CR (including R0 resection) and PR were observed in 40.7% and 59.3% of patients, respectively.

### Survival

The median follow-up period was 32.23 (2.7–198.2) months. At the last follow-up, two patients died at the 121 months, both of whom were in surgical group. Neither of the two patients died of seminoma. One of them died of pneumonitis, and the other died of myocardial infarction. One patient in each group experienced local recurrence, whereas one patient in surgical group had distant metastasis.

**Fig. 1:**
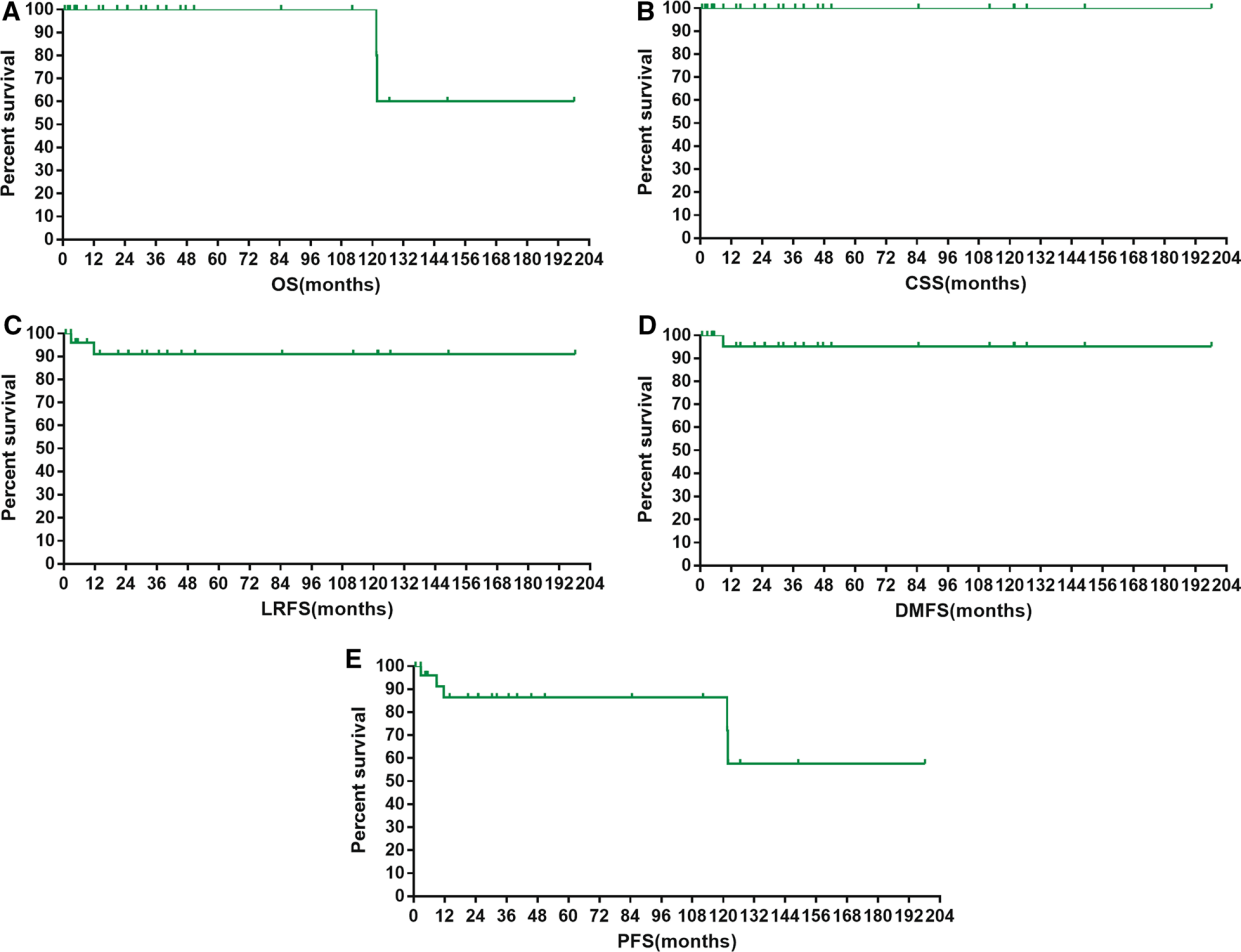
Overall survival (OS) (**A**), cancer-specific survival (CSS) (**B**), local regional-free survival (LRFS) (**C**), distant metastasis-free survival (DMFS) (**D**), and progression-free survival (PFS) (**E**) of patients with primary mediastinal seminoma

The median survival times were not achieved. The 5-year and 10-year survival rates were: OS, 100.0% and 100.0%; CSS, 100.0% and 100.0%; LRFS, 90.9% and 90.9%; DMFS, 95.2% and 95.2%; and PFS, 86.4% and 86.4%, respectively. The survival curves are shown in Fig. 1A–E.

**Fig. 2:**
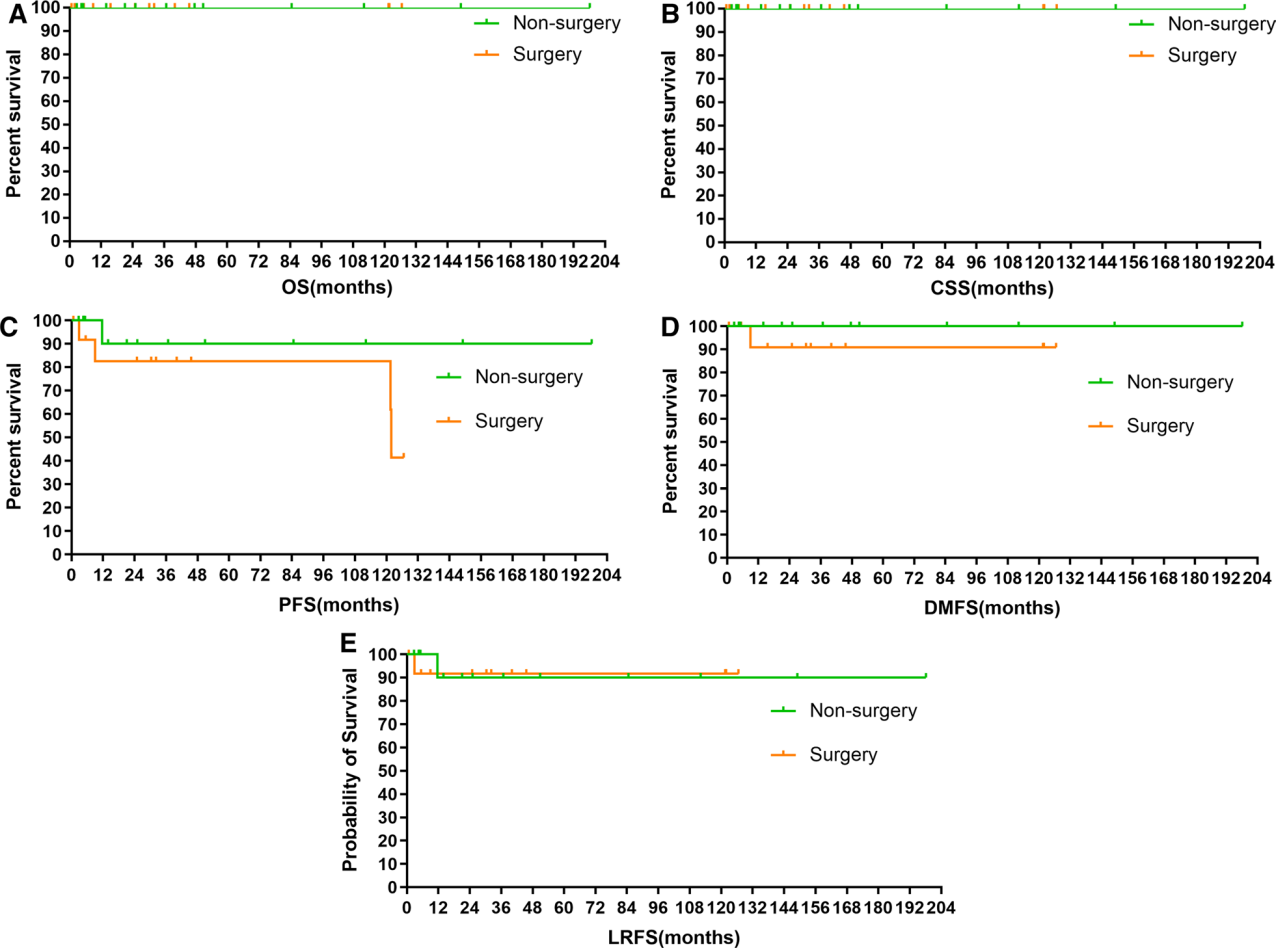
Comparisons of overall survival (OS) (**A**), cancer-specific survival (CSS) (**B**), local regional-free survival (LRFS) (**C**), distant metastasis-free survival (DMFS) (**D**), and progression-free survival (PFS) (**E**) between patients with and without surgery

There was no significant difference of OS (5-year 100% vs. 100%), CSS (5-year 100% vs. 100%), LRFS (5-year 91.7% vs. 90.0%, *p* = 0.948), DMFS (5-year 90.9% vs. 100.0%, *p* = 0.340) and PFS (82.5% vs. 90.0%, *p* = 0.245) between patients with and without surgery. The survival curves are plotted in Fig. 2A–C.

### Other prognostic factors

Similarly, as none of the patients died from seminoma at the last follow-up, univariate analysis of OS and CSS was not performed. On univariate analysis, patients with superior vena cava syndrome (SVCS) showed a better PFS. Sex was also associated with PFS.

**Fig. 3:**
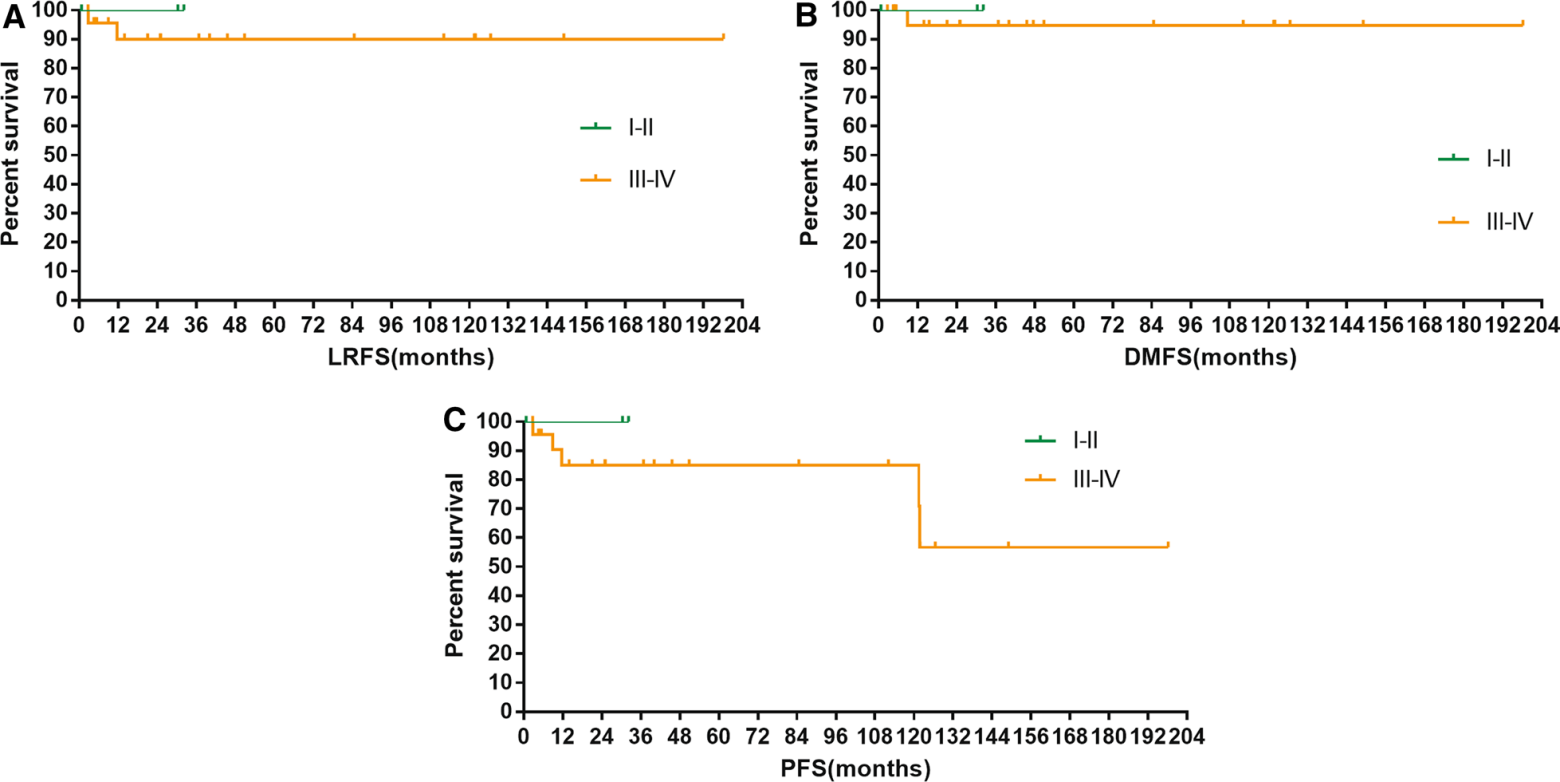
Local regional relapse-free survival (LRFS) (**A**), distant metastasis-free survival (DMFS) (**B**), and progression-free survival (PFS) (**C**) of patients according to Masaoka stage

For patients with Masaoka stage I–II and III–IV disease, 10-year LRFS were 100% and 90% (*p* = 0.651); 10-year DMFS were 100% and 94.7% (*p* = 0.746); and 10-year PFS were 100.0% and 87.0% (*p* = 0.574), respectively (Fig. 3A–C, Table 5). Patients who underwent R0 resection got better 10-year local regional relapse-free survival (100.0% vs. 86.3%, *p* = 0.305) without significant statistical differences.

**Table 5:** Prognostic factors

Prognostic factor	10-year LRFS (%)			10-year DMFS (%)			10-year PFS (%)		
	Yes	No	*p*	Yes	No	*p*	Yes	No	*p*
Male	90.8	100	0.838	100	0.00	0.000	90.8	0.0	0.002
Age, < 18 years	75.0	95.2	0.251	100	94.1	0.628	75.0	85.3	0.943
Symptoms	89.5	100	0.569	94.4	100	0.683	84.2	100	0.801
SVCS	100	84.8	0.238	100	92.3	0.433	100	77.8	0.011
Alcohol use	90.8	100	0.838	95.2	100	–	86.3	100	0.838
Smoking	100	88.7	0.472	100	94.1	0.628	100	83.0	0.620
GVI	93.3	83.3	0.352	100	83.3	0.114	93.3	66.7	0.307
Diameter, > 10 cm	100	83.6	0.194	90.0	100	0.294	90.0	83.6	0.407
LNM	100	88.1	0.419	80.0	100	0.704	80.0	88.1	0.602
DMPD	100	90.3	0.706	50.0	100	0.002	50.0	90.3	0.097
Masaoka stage III–IV	90.0	100	0.651	94.7	100	0.746	87.0	100	0.574
R0 resection	100	86.3	0.305	87.5	100	0.202	87.5	86.3	0.715
Radiotherapy	93.3	87.5	0.797	92.3	100	0.433	85.6	87.5	0.236
Chemotherapy	90.5	100	0.755	95.0	100	0.823	100	85.7	0.188

*DMFS* distant metastasis-free survival, *DMPD* distant metastasis at the primary diagnosis, *GVI* great vessel invasion, *LNM* lymph node metastasis, *LRFS* local–regional relapse-free survival, *PFS* progression-free survival, *SVCS* superior vena cava syndrome

## Discussion

Mediastinal seminomas are difficult to depict because of their rarity. In this study, we investigated a relatively large number of patients with primary mediastinal seminomas. Seminomas usually show slow growth and have an invasive course, although the disease is often asymptomatic at onset. The absence of symptoms leads to disease diagnosis at a more advanced stage because most patients do not seek medical attention until symptom manifestation. The most common symptoms in our study are consistent with those observed in previous studies, with chest pain (14.3–44%), cough (14.3–38%), and dyspnea (14.3–38%) being the top three symptoms [3, 8, 9]. During diagnosis, only 11.1% of the patients in our study were asymptomatic, which is almost equivalent to that reported in previous studies (6–40%) [3–5, 8].

Due to their slow growth, most seminomas are bulky when diagnosed. The median maximum diameter of the primary tumor (9.9 cm) is consistent with previous findings (8–12 cm) [3, 8, 9]. The tumor may extend to the mediastinum, leading to compression of adjacent structures and invasion, especially into the great vessels in the mediastinum, such as the SVC and aorta. In our study, 37% of patients were found to have SVCS, which is consistent with the findings of previous reports (10–57%) [3, 4, 8, 9]. However, there was no mention of invasion into the aorta in these previous studies, which might cause difficulties in operation.

In previous small-scale studies and case reports, the 5- and 10-year OS of patients with primary mediastinal seminomas ranged from 87 to 100% and from 75 to 100%, respectively [3, 4, 8–10]. One study also showed a 5-year LRFS of 82.1%. These findings are consistent with our findings. None of the patients in our study died of seminoma at the last follow-up. Despite the cumulative 10-year risk of testicular malignancy of 10.3% after a diagnosis of extragonadal germ-cell tumor [11], no study patient showed testicular invasion or metastasis at the last follow-up. Thus, the prognosis of patients with primary mediastinal seminoma was generally good. Local relapse and distant metastasis were low after treatment. In 2015, our institution carried out a retrospective study to investigate the clinical characteristics and outcomes of patients with primary malignant mediastinal non-seminomatous germ-cell tumor [12]. Compared with that study, our study achieved better OS (100% vs. 49.2%) and PFS (100% vs. 32.8%). The result of the comparison is also consistent with that of previous reports [3, 10, 13]. A series of small, combined studies also compared the OS of the two different types of mediastinal germ-cell carcinoma. The studies showed that patients with seminomas achieved a better 5-year OS than those with non-seminomas (87.0–100% vs. 36.7–83.0%), although not all the studies showed statistical significance due to limited sample size.

On basis of the upper studies, various treatments for mediastinal seminoma aim for complete cure rather than just symptom relief. Theoretically, surgery is the predominant treatment for most of the malignancies, such as testicular seminoma. For patients with mediastinal seminoma, R0 resection is difficult to perform because of tumor invasion into adjacent mediastinal structures, with only 12.5% of patients undergoing such procedure in previous studies [8]. In our study, 51.9% received surgery and 33.3% of patients underwent R0 resection. The postoperative disease control rate was consistent with that in previous study (90–100%) [14]. However, we found that patients without surgery, even though there were more patients with poor performance score (100% vs. 76.9%), more patients (100% vs. 76.9%) with adjacent structures invasion, more patients with great vessel (100% vs. 46.2%) especially aorta invasion (78.6% vs. 30.8%) in this group, got non-inferior OS, CSS, PFS, LRFS and DMFS compared with that in surgery group, probably because of a favorable prognosis and sensitivity to chemoradiotherapy.

Generally, most patients undergo chemotherapy receive BEP, as do patients with testicular seminoma. Mediastinal seminoma also demonstrates a high sensitivity to chemotherapy. In this study, 92.6% of patients received chemotherapy, with response rates of 95.0%, which is consistent with those (83–90%) reported previously [14]. However, whether chemotherapy could affect the local recurrence or distant metastasis is still uncertain because of the limited number of patients and the limited number of events.

Nearly 60% of the patients received radiation and the response rate is 100%. The results reached agreement with those in previous studies (80–100%) [14]. Also, radiation might decrease local recurrence. Unlike routine chemotherapy regimens, radiation is delivered in different doses (25.2–56 Gy). In one study, the patients received 2 Gy × 30 fractions [9]. Comparing this finding with our finding revealed no significant difference in either survival or response rate, which may be due to high chemoradiosensitivities. Furthermore, a dose of 45 Gy might be a reasonable choice when considering the patient’s quality of life as well as reducing the toxicities in long term. Referring to testicular seminoma, different doses should be prescribed according to the resection range and residual disease.

In this study, the toxicities in both groups were tolerable. Hair loss was the most common toxicity probably because of the use of VP-16. Due to the special location of this disease and the delivery of bleomycin, we monitored for radiation-induced pneumonitis (RP). Only five patients were diagnosed with grade 1 RP, and no severe RP was observed because of both the reasonable radiation dose and the utilization of modern radiation techniques. Although there have been no cardiac-related adverse events documented, a long-term follow-up for cardiac toxicities is necessary because the heart is one of the adjacent organs.

To provided more evidence for this disease with sporadic morbidity, we also summarized some characteristics and our comments as following.

Seminomas mostly occur in men, usually young patients. In our study, the median age was 28 years, which is consistent with that in previous reports (28–34 years) [3, 8]. There have been only a few case reports on female patients [2, 6, 8]. Our study included a woman aged 44 years with pericardial invasion. She underwent R0 resection and postoperative chemoradiotherapy and survived until the last follow-up (8.9 months) with pleural metastasis. Although data showed inferior PFS and DMFS, it is difficult to appropriately determine the relationship between sex and survival rates.

The results regarding the IHC characteristics of patients varied. The positivity rate for PLAP was 70.7% in a previous study [14]. In our study, all 18 patients who underwent the PLAP test were PLAP-positive. The positivity rates of OCT3/4, SALL4, and CD117 were also high. This suggests that PLAP could be the most remarkable marker for mediastinal seminoma.

Previous studies have reported elevated β-hCG levels in 0–85.7% of patients with primary mediastinal seminoma [3–5, 8] In our study, 51.8% of patients showed increased β-hCG levels, which is similar to the findings of a previous study [9]. Such elevated levels might be attributed to tumor enlargement. Meanwhile, serum LDH was not a typical marker of the disease, which is consistent with previous results [3, 9].

In this study, the perivascular station was the most common invasion site (100%), followed by the para-aortic station (70.4%) and subaortic station (51.9%). These results are similar to those of previous studies, which found that mediastinal seminomas are usually located in the anterior mediastinum and in front of the aorta [9]. The other common invasion sites were the bilateral lower paratracheal station (40.7%, 40.7%) and bilateral upper paratracheal station (40.7%, 40.7%).

There is no established staging system for mediastinal seminomas, and the testicular seminoma staging system cannot be used either. However, mediastinal seminomas seem to share some homogeneous characteristics with thymic neoplasms. Both are prevascular tumors and occur in the anterior mediastinum, both are with rare lymph node metastasis and both are associated with a good prognosis. Based on these common aspects, we adopted the Masaoka staging system, which is widely used for thymic neoplasms, to evaluate the status of mediastinal seminomas [6, 9]. We found that 88.9% of patients were diagnosed as having Masaoka stage III–IV disease. Lymph node metastasis and distant metastasis, on the other hand, are not as common as great vessel invasion. A previous study found that lymph node metastasis occurred in 2.6–38% of patients [8]. Although these findings indicate no significant differences in the prognosis of patients with different Masaoka stages, we found a trend that patients with stage I–II disease exhibited higher DMFS and PFS.

The strong point of our study is multifold. First, to our knowledge, this is the largest study focusing on mediastinal seminomas. Most of the patients in this study underwent modern radiation methods and could represent modern real-world data. Our findings could provide a basis for future treatment delivery in patients with primary mediastinal seminomas. Secondly, we found that compared with surgery, non-surgery treatment brought non-inferior results in both efficacy and safety in patients with mediastinal seminoma invading adjacent organs especially great vessels, which have not been mentioned in previous studies. Thirdly, we also described the common location of this disease according to mediastinal lymph node system. Finally, we first borrowed Masaoka stage from thymoma to depict the stage of seminomas and declared an association between PFS, DMFS and Masaoka stage.

As a retrospective study, there is also some limitations. First, different treatment regimens comprising various therapeutic agents were used with no definite guidelines. Secondly, certain components of the IHC test were not possible in some samples because of the long investigation period and deterioration in storage conditions. Thirdly, we did not have enough time to evaluate late toxicities due to the limited follow-up period. Finally, it is hard to draw a definite conclusion to choose a best therapeutic method among R0 resection, R1/R2 resection plus chemoradiotherapy and non-surgery treatment for the limited number of patients.

## Conclusions

In conclusion, this study revealed that mediastinal seminomas were frequently diagnosed as large tumors, were in the anterior mediastinum and prevascular region, and always invaded the great vessels. Although these invasions increase the difficulties to perform operation, surgical treatments did not affect the survivals and progressions. Our study also used different modes of combined chemoradiotherapies, and all of them achieved favorable results with moderate toxicities. R0 resection and radiotherapy might be helpful to avoid local regional relapses.

## Data Availability

The data would be obtained by sending email to the corresponding authors for academic research.
